# Machine Learning-Based CT Radiomics Analysis for Prognostic Prediction in Metastatic Non-Small Cell Lung Cancer Patients With *EGFR*-T790M Mutation Receiving Third-Generation EGFR-TKI Osimertinib Treatment

**DOI:** 10.3389/fonc.2021.719919

**Published:** 2021-09-29

**Authors:** Xin Tang, Yuan Li, Wei-feng Yan, Wen-lei Qian, Tong Pang, You-ling Gong, Zhi-gang Yang

**Affiliations:** ^1^ Department of Radiology, West China Hospital, Sichuan University, Chengdu, China; ^2^ Department of Thoracic Oncology and State Key Laboratory of Biotherapy, Cancer Center, West China Hospital, Sichuan University, Chengdu, China

**Keywords:** lung cancer, EGFR-T790M, osimertinib, radiomics, prognostic prediction

## Abstract

**Background and Purpose:**

As a third-generation *EGFR* tyrosine kinase inhibitor (TKI), osimertinib is approved for treating advanced non-small cell lung cancer (NSCLC) patients with *EGFR*-T790M mutation after progression on first- or second-generation EGFR-TKIs such as gefitinib, erlotinib and afatinib. We aim at exploring the feasibility and effectiveness of using radiomic features from chest CT scan to predict the prognosis of metastatic non-small cell lung cancer (NSCLC) patients with *EGFR*-T790M mutation receiving second-line osimertinib therapy.

**Methods:**

Contrast-enhanced and unenhanced chest CT images before osimertinib treatment were collected from 201 and 273 metastatic NSCLC patients with *EGFR*-T790M mutation, respectively. Radiomic features were extracted from the volume of interest. LASSO regression was used to preliminarily evaluate the prognostic values of different radiomic features. We then performed machine learning-based analyses including random forest (RF), support vector machine (SVM), stepwise regression (SR) and LASSO regression with 5-fold cross-validation (CV) to establish the optimal radiomic model for predicting the progression-free survival (PFS) of osimertinib treatment. Finally, a combined clinical-radiomic model was developed and validated using the concordance index (C-index), decision-curve analysis (DCA) and calibration curve analysis.

**Results:**

Disease progression occurred in 174/273 (63.7%) cases. CT morphological features had no ability in predicting patients’ prognosis in osimertinib treatment. Univariate COX regression followed by LASSO regression analyses identified 23 and 6 radiomic features from the contrast-enhanced and unenhanced CT with prognostic value, respectively. The 23 contrast-enhanced radiomic features were further used to construct radiomic models using different machine learning strategies. Radiomic model built by SR exhibited superior predictive accuracy than RF, SVR or LASSO model (mean C-index of the 5-fold CV: 0.660 *vs.* 0.560 *vs*. 0.598 *vs*. 0.590). Adding the SR radiomic model to the clinical model could remarkably strengthen the C-index of the latter from 0.672 to 0.755. DCA and calibration curve analyses also demonstrated good performance of the combined clinical-radiomic model.

**Conclusions:**

Radiomic features extracted from the contrast-enhanced chest CT could be used to evaluate metastatic NSCLC patients’ prognosis in osimertinib treatment. Prognostic models combing both radiomic features and clinical factors had a great performance in predicting patients’ outcomes.

## Introduction

Lung cancer is the second most common tumor in both men and women and the first leading cause of cancer-related mortality worldwide (1). Non-small cell lung cancer (NSCLC) accounts for over 80% of patients with lung cancer (2). Although NSCLC patients of early stage can achieve satisfactory survival outcomes through definitive treatment, the prognosis of advanced NSCLC patients remains poor.

The recent 20 years have witnessed the emergence and development of tyrosine kinase inhibitors (TKIs) in treating advanced lung cancer (3). Among them, osimertinib, as the first third-generation TKI agent, attracted the most attention. Osimertinib has been approved for treating NSCLC patients with epidermal growth factor receptor (*EGFR*) T790M mutation after progression on first- or second-generation EGFR-TKIs such as gefitinib, erlotinib and afatinib (4). Despite the promising therapeutic efficacy of osimertinib, it cannot be neglected that even among NSCLC patients with *EGFR*-T790M, not all of them can achieve satisfactory clinical benefit from this agent. However, currently, there are only a few studies that explore factors related to the therapeutic efficacy of osimertinib treatment. Several studies explored factors that can potentially affect the treatment efficacy of osimertinib in NSCLC patients. Clinical factors including male gender, smoking history, high N stage and poor performance score were associated with poor outcomes in osimertinib treatment (5–8).

As a rapidly emerging technique, radiomic analysis attracted increasing attention in recent years for its advantages in providing objective and quantifiable imaging information (9). Numerous studies proved the feasibility and effectiveness of radiomics in distinguishing begin and malignant disease and predicting cancer stages, pathological findings, genomic alterations, patients’ prognosis and etc (9). Several studies reported that radiomic features were capable of predicting certain mutations of the *EGFR* gene (10, 11). Of note, some researchers also showed that radiomic features had a great potential in predicting the clinical outcomes of NSCLC patients with sensitive *EGFR* mutation (19del/L858R) receiving first- or second-generation EGFR-TKIs as first-line targeted therapy (12–14). However, there is still a lack of radiomic research on third-generation EGFR-TKI. Thus, it is reasonable to speculate that radiomics may also have the ability in predicting the prognosis of third-generation EGFR-TKI osimertinib.

The aim of our study is to investigate the value of radiomic features extracted from chest computed tomography (CT) in predicting the prognosis of second-line osimertinib treatment in advanced NSCLC patients with *EGFR*-T790M mutation.

## Materials and Methods

### Patients

This study was approved by the Ethics Committee of West China Hospital and individual consent for this retrospective analysis was waived. A total of 273 metastatic NSCLC patients harboring *EGFR*-T790M mutation and without radical surgery from 2017-2021 in West China Hospital were included. All patients were treated with either gefitinib 148 (54.2%), erlotinib 45 (16.5%), icotinib 73 (26.7%) or afatinib 7 (2.6%) as first-line *EGFR*-TKI treatment and developed disease progression. After that, all cases were administrated with osimertinib (80 mg orally once daily) following the confirmation of *EGFR*-T790M mutation from either the blood sample or tissue sample. Before osimertinib therapy, the total cohort received an unenhanced chest CT scan, while 201 (73.6%) also had contrast-enhanced CT simultaneously. Patients without accessible CT images or with CT images of poor quality were excluded from this study.

### CT Imaging Parameters

All included patients were performed with the following five CT scanners: SOMATOM Definition, SOMATOM Definition Flash and SOMATOM Definition AS+(Siemens Medical Systems); uCT 780 (UIH Medical Systems) and Revolution CT (GE Medical Systems). The acquisition parameters of CT were as follows: tube voltage: 100 -120 kV; tube current: 100 - 650 mA; rotation time: 0.5s; pitch: 1.0–1.5; collimation: 0.5–0.625mm; pixel size: 0.535 × 0.535 mm^2^ to 0.976 × 0.976 mm^2^; matrix: 512 x 512; slice thickness: 5 mm; lung window setting: width, 1200 HU; level, −600 HU; mediastinal window setting: width, 350 HU; level, 50 HU. Contrast-enhanced CT was performed after the intravenous injection of the nonionic contrast agent (80-100ml) at a flow rate of 3ml/s. The arterial phase was obtained after 30s following the injection of contrast agents.

### The Volume of Interest Delineation, Data Pre-Processing and Feature Extraction

The VOI delineation of contrast-enhanced and unenhanced chest CT was performed by two radiologists, separately. Lung tumors were manually delineated on all slices using the open-source software 3D slicer (Version 4.10.2) (15). To standardize the voxel spacing across the cohort, all CT voxels were resampled to 1x1x1 mm^3^ before feature extraction. A bin width of 25 Hounsfield units (HU) was used for texture features. Eventually, 1223 radiomic features were obtained and could be classified into three categories including 14 shape, 234 first order and 975 texture features, with and without Wavelet and Laplacian of Gaussian (LoG) filtering. Texture features included gray-level co-occurrence matrix (GLCM), gray-level run length matrix (GLRLM), gray-level difference matrix (GLDM), gray-level size zone (GLSZM) and neighborhood gray tone difference Matrix (NGTDM).

Combat algorithm was performed for feature harmonization and the adjustment of batch effects (https://github.com/Jfortin1/ComBatHarmonization). The Z-score transformation was used for radiomic features normalization. Interclass correlation coefficient (ICC) was applied to assess the consistency of radiomic features between radiologists. Only radiomic features with ICC >0.9 were included in further analyses.

### Endpoint

The endpoint of this study was progression-free survival (PFS) which was defined as the interval from the beginning of second-line osimertinib treatment to disease progression according to the Response Evaluation Criteria in Solid Tumors (RECIST) 1.1 criteria.

### Statistical Analyses

Univariate COX regression was performed to preliminarily analyze the ability of each radiomic feature in predicting the PFS of the second-line osimertinib treatment. To shrink the dimensionality of radiomic features, the least absolute shrinkage and selection operator (LASSO) COX regression *via* 10-fold cross-validation based on minimum criteria was also carried out.

Machine learning-based analyses including random forest (RF), support vector machine (SVM), stepwise regression (SR) as well as LASSO COX were conducted to build the optimal radiomic model. We applied the randomForestSRC, survivalsvm, MASS and glmnet R packages to perform the RF, SVM, SR and LASSO COX analysis, respectively. The randomForestSRC package contains Breiman’s random forest function. 1000 trees were used to generate the random forest models while other parameters were set as default. The survivalsvm package embeds an SVM algorithm specifically for survival data. In the SVM analysis, the “regression” approach raised by Van Belle et al. was utilized (16). In the SR algorithm, we chose “both” as the mode of stepwise search, which means that both the backward and forward direction for variable selection were applied.

A 5-fold cross-validation (CV) strategy was performed. Specifically, the total cohort was randomly split into five sub-cohorts. Four of the five sub-cohorts were used for model training by different machine learning methods while the remaining one sub-cohort was used for model testing. This process was repeated five times until all combinations were covered. Then machine learning method with the highest mean concordance index (C-index) was chosen to build the radiomic model.

A radiomic and radiomic model integrating both clinical predictive factors and radiomic features was also developed. In terms of model evaluation, C-index was used to reflect the predictive accuracy of the model. Besides, decision curve analysis (DCA) and calibration curves were used to evaluate the clinical benefit and the goodness-of-fit of the model. Statistical analyses were conducted using R software (V 4.0.5).

## Results

### Patients’ Characteristics

Baseline factors of the total 273 patients are shown in **Table 1**. Overall, 106 (38.8%) male and 167 (61.2%) female patients were included. At the end of follow-up, disease progression developed in 174/273 (63.7%) cases. The median PFS of the total patients was 13.3-Mo (95%CI: 11.8-13.9 Mo).

**Table 1:** Baseline factors of the total 273 patients receiving second-line osimertinib therapy.

Baseline Factor	Number (Percentage)
**Age (Year)**	
** Median (Interquartile range)**	57.0 (51.0-68.0)
** <60**	145 (53.1%)
** ≥60**	128 (46.9%)
**Smoking History**	
** Without**	218 (79.9%)
** With**	55 (20.1%)
**Gender**	
** Male**	106 (38.8%)
** Female**	167 (61.2%)
**Performance Score**	
** 0**	188 (68.9%)
** 1**	72 (26.4%)
** ≥2**	13 (4.8%)
**T stage prior Osimertinib**	
** T1-2**	78 (28.6%)
** T3-4**	195 (71.4%)
**N stage prior Osimertinib**	
** N0-1**	105 (38.5%)
** N2-3**	168 (61.5%)
**M stage prior Osimertinib**	
** M1a**	65 (23.8%)
** M1b**	25 (9.2%)
** M1c**	183 (67.0%)
**Stage prior Osimertinib**	
** IVA**	90 (33.0%)
** IVB**	183 (67.0%)
**First-line TKI**	
** Gefitinib**	148 (54.2%)
** Erlotinib**	45 (16.5%)
** Icotinib**	73 (26.7%)
** Afatinib**	7 (2.6%)
**Chest CT**	
** Contrast enhanced CT**	201 (73.6%)
** Unenhanced CT**	273 (100.0%)

TKI, Tyrosine Kinase Inhibitor.

### The Prognostic Value of Clinical Factors and CT Morphological Features in Osimertinib Treatment

**Figure 1:**
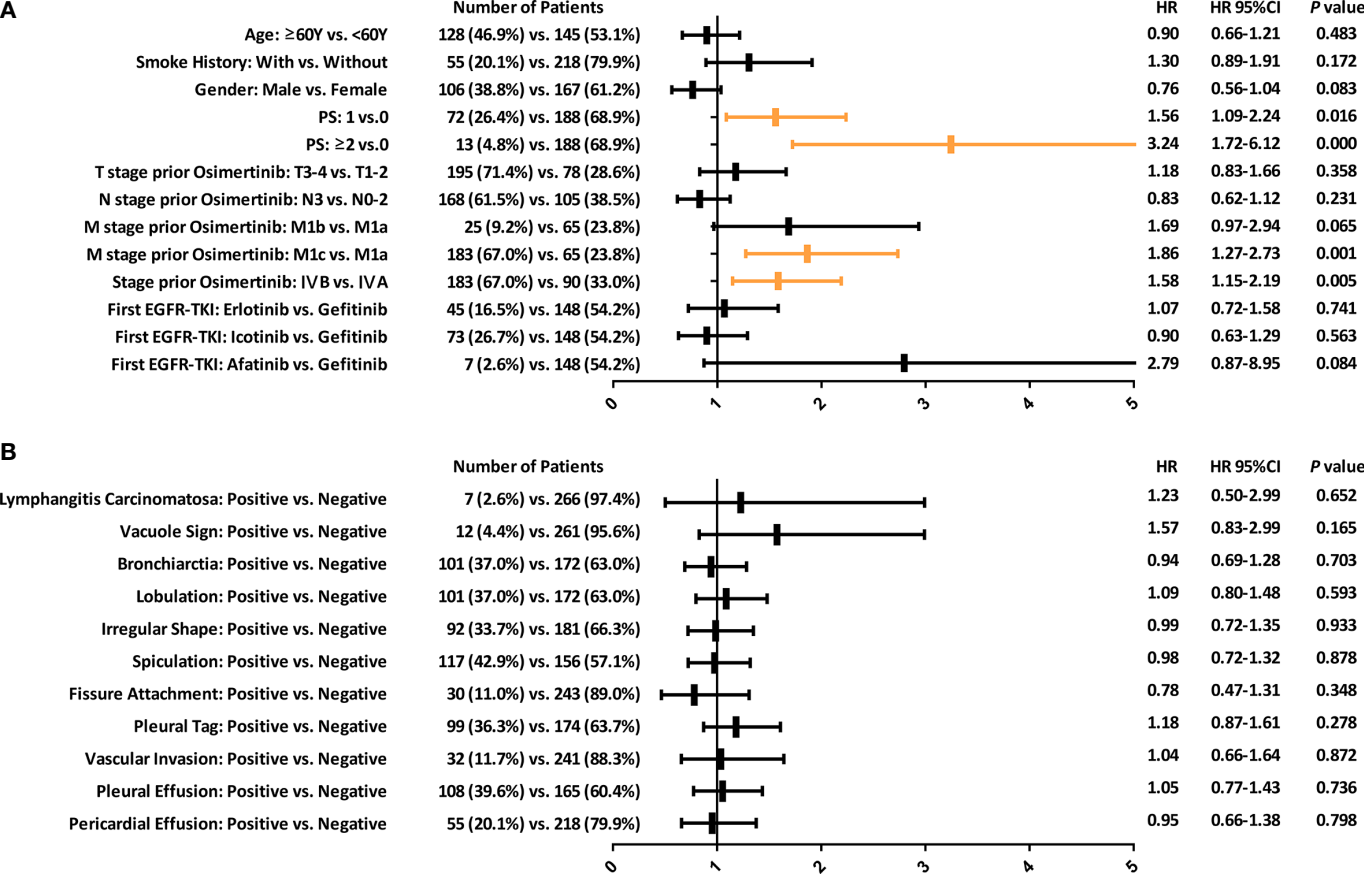
Forest plots depicting the prognostic value of various clinical factors **(A)** and morphological CT features **(B)** in predicting the PFS of patients with *EGFR*-T790M mutation after second-line osimertinib therapy. PFS, Progression-free survival.

The significance of clinical factors (**Figure 1A**) and CT morphological features (**Figure 1B**) in predicting the PFS of the second-line osimertinib treatment was investigated. Clinical factors including high performance score (PS), M1c stage and stage IV was associated with poor prognosis. Multivariate analyses confirmed that PS=2 (HR, 95%CI: 2.86, 1.52-5.40) and M1c stage (HR, 95%CI: 1.83, 1.25-2.70) were independent predictors of PFS.

The predictive ability of CT morphological features was limited (**Figure 1B**). All morphological features were unrelated to the therapeutic efficacy of second-line osimertinib treatment in NSCLC patients harboring *EGFR*-T790M mutation.

### The Prognostic Value of Radiomic Features Extracted From Contrast-Enhanced and Unenhanced Chest CT

**Figure 2:**
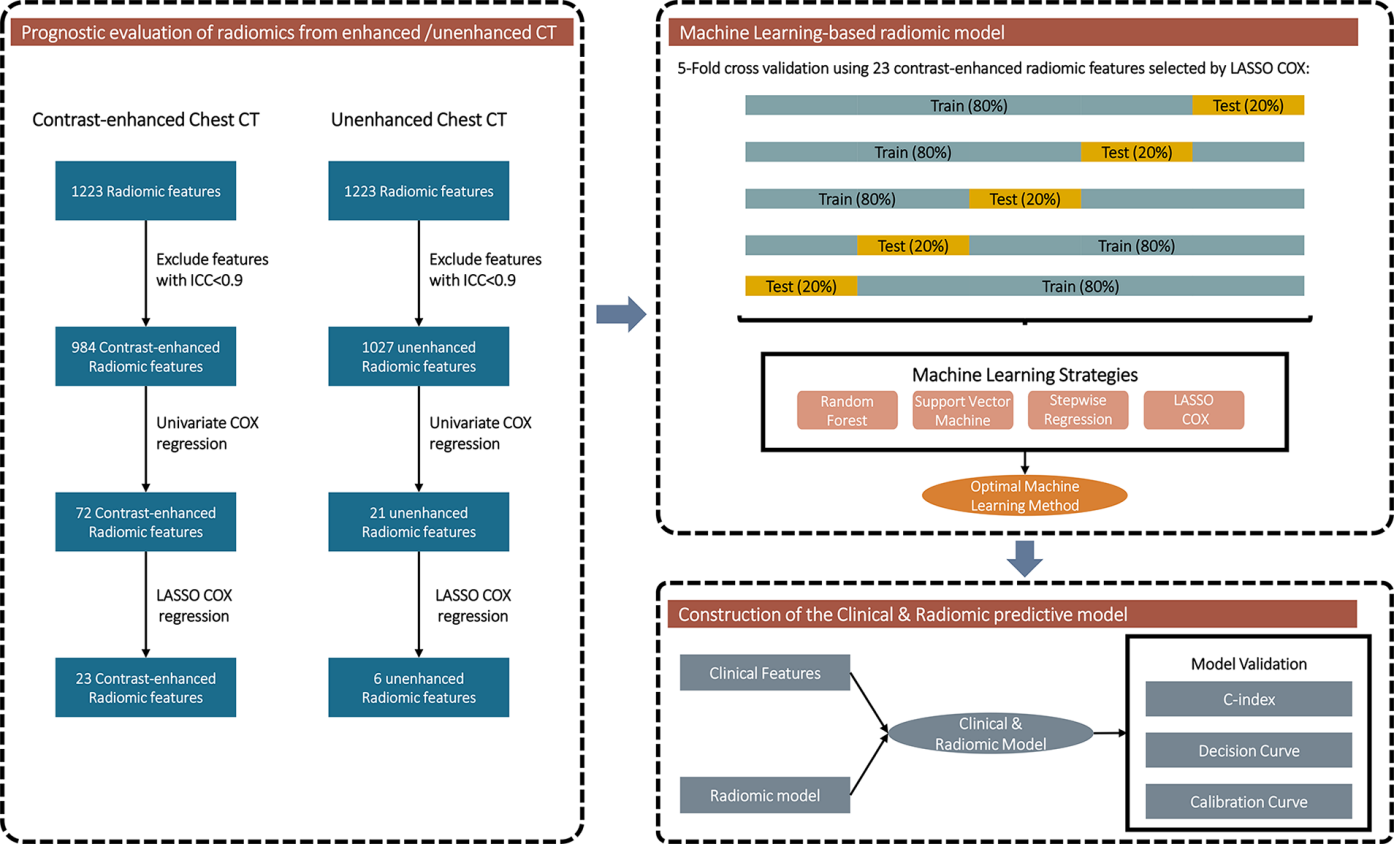
Flowchart exhibiting the general design of the prognostic analyses of the radiomic features.

**Figure 2** exhibits the overall design of the prognostic analyses of the radiomic features. Radiomic features with ICC >0.9 were included in prognostic analyses after combat adjustment. In aggregate, 984/1223 (80.5%) and 1027/1223 (84.0%) features from the contrast-enhanced and unenhanced chest CT were eligible, respectively.

**Figure 3:**
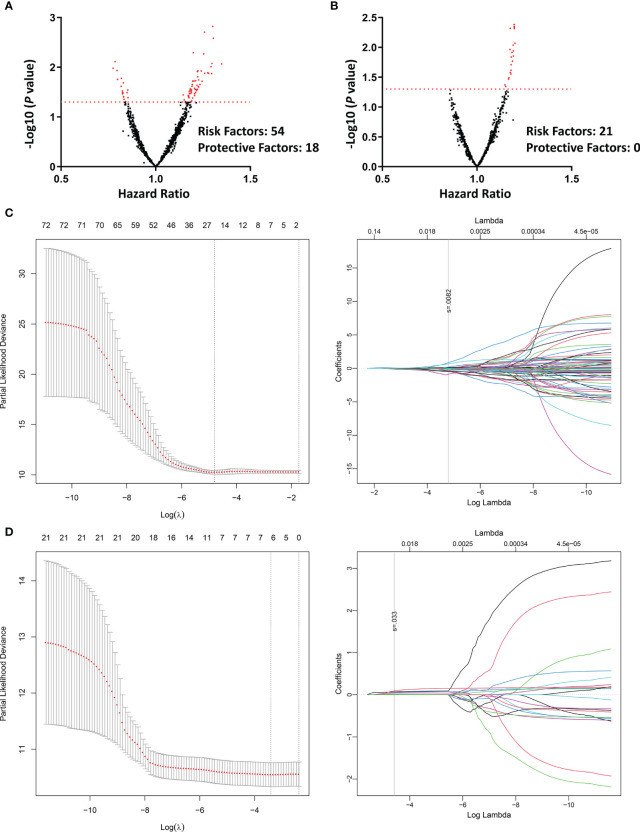
Univariate COX regression and LASSO regression assessing the prognostic value of different radiomic features in predicting the efficacy of second-line osimertinib therapy. **(A, B)**, Volcano plots reflecting each radiomic feature’s value in predicting PFS [**(A)**, contrast-enhanced phase; **(B)**, unenhanced phase]; **(C, D)**, LASSO COX regression based on radiomics feature from the contrast-enhanced **(C)** and unenhanced **(D)** chest CT. PFS, Progression-free survival; LASSO, Least absolute shrinkage and selection operator.

Univariate COX regression was applied to assess the prognostic value of each radiomic feature. Totally, 72 features from contrast-enhanced CT could predict PFS (**Figure 3A**), whereas only 21 features from unenhanced CT were associated with PFS (**Figure 3B**). LASSO regression was also conducted to further eliminate redundant radiomic features. The results turned out that 23 contrast-enhanced radiomic features were predictors of patients’ prognosis (**Figure 3C**). In sharp contrast, merely 6 features from the unenhanced CT were retained in the LASSO regression selection (**Figure 3D**). The correlation of the 23/6 features is shown in **Figure S1**. A moderate correlation was found between some of the radiomic features. Taking together, the above analyses implied that radiomic features from the contrast-enhanced chest CT were promising in predicting disease progression of the second-line osimertinib treatment, whereas the prognostic value of unenhanced CT radiomic features was weak.

### Prognostic Model Based on Contrast-Enhanced CT Radiomic Features

**Figure 4:**
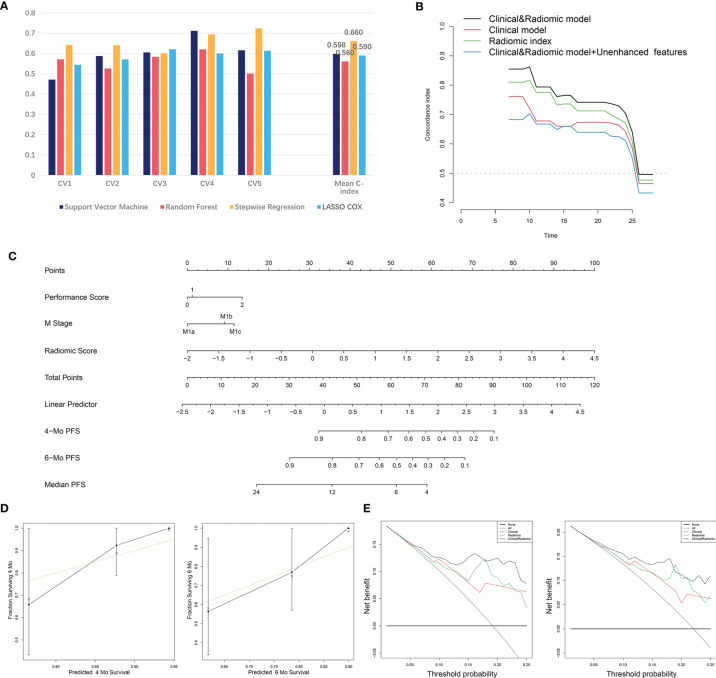
The development and validation of the radiomic and clinical and radiomic model. **(A)** Comparison of C-index of different machine learning methods in the 5-fold cross-validation. **(B)** C-index of the radiomic, clinical and clinical and radiomic model at distinct time points. **(C)** Visualizing of the clinical and radiomic model by nomogram. **(D)** Validation of the clinical and radiomic model by DCA. **(E)** Validation of the clinical and radiomic model by calibration curve analysis. C-index, Concordance index; DCA, Decision curve analysis.

To further evaluate the integrated prognostic effect of radiomic features from the contrast-enhanced CT, a radiomic-based prognostic model was developed using the 23 features selected by the LASSO regression. Machine learning strategies including RF, SVM, SR and LASSO regression with 5-fold CV were performed (**Figure 2**). The C-index of each predictive model was calculated (**Figure 4A**). SR displayed stronger predive power against RF, SVM and LASSO methods (mean C-index of the 5-fold CV: 0.660 *vs*. 0.560 *vs*. 0.598 *vs*. 0.590). The highest predictive accuracy (C-index: 0.724) of the SR method was achieved in model 5 which was selected as the final radiomic model. The included radiomic features based on the SR method are shown in **Table 2**.

**Table 2:** Stepwise COX regression predicting the PFS of the second-line osimertinib treatment.

Radiomic Features			HR	95%CI HR	*P* value
log-sigma-1-0-mm-3D	glcm	Cluster Shade	0.58	0.45-0.76	<0.001
log-sigma-1-0-mm-3D	glcm	Difference Variance	3.10	1.40-6.87	0.005
log-sigma-1-0-mm-3D	glrlm	Gray Level Variance	0.20	0.09-0.45	0.000
log-sigma-1-0-mm-3D	ngtdm	Strength	2.30	1.53-3.45	0.000
wavelet-HLL	glcm	Imc2	1.31	0.92-1.87	0.098
wavelet-LHL	glcm	Imc2	0.50	0.31-0.78	0.003
wavelet-LHL	glszm	Gray Level Non Uniformity Normalized	0.50	0.32-0.79	0.003
wavelet-LHL	ngtdm	Contrast	0.67	0.42-1.09	0.095
original	glcm	Inverse Variance	0.74	0.54-1.02	0.064

HR, Hazard ratio; CI, Confidence interval; PFS, Progression-free survival.

### The Development and Validation of the Clinical and Radiomic Model

We then attempted to integrate clinical factors and radiomic model together and established a clinical and radiomic model. Clinical factors including PS and M stage as well as the radiomic model were used for the model construction. The final clinical and radiomic model harbored a higher C-index at distinct time points compared to the clinical or radiomic model alone, and an integrated C-index of 0.755 (**Figure 4B**). Next, we attempted to add the 6 unenhanced features with predictive value into the model to see if the predictive accuracy of the model could be further improved. Yet, after we incorporated the 6 unenhanced features into the model, the C-index of the model did not rise but fall (from 0.755 to 0.733). Besides, C-index at different time points also revealed that incorporating the unenhanced features into the model did not improve its predictive performance (**Figure 4B**). These results supported that the predictive significance of unenhanced features was limited. The final model was visualized by nomogram (**Figure 4C**). Besides, the DCA reflected satisfactory clinical utility of the model, which was better than the clinical or radiomic model alone (**Figure 4D**). In addition, the calibration curves also indicated moderate goodness-of-fit of the clinical and radiomic model (**Figure 4E**).

## Discussion

Advanced NSCLC is an incurable stage of disease with generally poor survival outcomes. The emergence of osimertinib, a third-generation TKI, has given new hope to patients suffering from this disease (17). Here, we initiatively investigated the value of radiomic and morphological features from chest CT scan in predicting the prognosis of patients receiving second-line osimertinib therapy. The results turned out that, the association between morphological CT features and patients’ prognosis was weak. The prognostic ability of radiomic features extracted from the unenhanced chest CT scan was also unsatisfactory. In sharp contrast, radiomic features from the contrast-enhanced chest CT showed great potential in predicting the therapeutic efficacy of the second-line osimertinib treatment. The adding of contrast-enhanced radiomic features to the clinical model can obviously increase the predictive accuracy.

TKIs have been widely used in treating advanced NSCLC patients with *EGFR* mutation (3). However, treatment failure will inevitably occur in almost all cases receiving TKIs. One major reason driving the resistance to the first- or second-generation TKI agents is the development of secondary *EGFR*-T790M mutation (18). According to the results from the phase III clinical trial, the third-generation TKI, osimertinib, showed superior efficacy against platinum-based chemotherapy for NSCNC patients with *EGFR*-T790M mutation after the failure of first-line TKI therapy (4). Besides, as one of the most common sites for NSCLC metastasis, brain metastasis occurs in approximately 30-50% of NSCLC patients (19). Yet, the blood-brain barrier penetration ability of the first- and second-generation TKI agents is very limited (20, 21). In comparison, osimertinib can more easily pass through the blood-brain barrier and has a better therapeutic effect on brain metastases (22).

Despite that osimertinib shows great superiority compared to the first- or second-generation TKIs. Heterogeneity of therapeutic response of osimertinib treatment still exists among NSCLC patients. Thus, it is of great importance to explore the potential prognosticators of the osimertinib treatment and to identify the most suitable cases for such therapy, which is the key to increase the cost-effectiveness of the osimertinib-related treatment decision-making. At present, only a few researchers had explored clinical factors that could potentially affect the therapeutic efficacy of the osimertinib treatment. Factors including smoking history, high N stage and poor performance score were related to an unfavorable prognosis in the osimertinib therapy (5–8). In the current study, smoking history and N stage were not associated with patients’ outcomes in osimertinib treatment. A possible explanation for this was that only metastatic NSCLC patients without radical surgery were included in this study. Resultingly, in this aggressive cohort, the prognostic values of smoking history and N stage were diluted. The prognostic value of other clinical factors such as age and gender remain inconsistent among various researches. There are studies showing that young age is related to a favorable (23), unfavorable (24) prognosis or no prognostic significance (25), and the same controversial reports for gender (5, 25). Besides, the PFS of the first-line TKIs treatment may also impact the efficacy of the sequential osimertinib therapy (26). Pre-treatment genomic aberration, such as loss of T790M, was also a predictor of rapid disease progression in second-line osimertinib therapy (27, 28).

Radiomics is an emerging technique that has been applied to various research fields (9). The advantage of radiomics analysis is its convenience and non-invasiveness in clinical use. Recently, the significance of radiomics the oncological researches has gained particular attention. Radiomics also had a great potential in predicting the prognosis of NSCLC patients treated with TKIs. Early in 2014, Yousun and colleagues found that quantitative CT variables are capable of predicting the tumor response to the TKI and concurrent chemoradiation therapy (29). In 2018, another research group reported that CT texture analysis could act as a prognosticator in metastatic lung adenocarcinoma patients treated with either erlotinib or gefitinib (12). Likewise, CT-based radiomic signatures were also associated with the prognosis of ALK-positive NSCLC patients receiving crizotinib (30). Thus, it is reasonable to speculate that radiomics may also be used in the prognostic evaluation of NSCLC patients with *EGFR*-T790M mutation receiving second-line osimertinib.

In this study, we explored if radiomic features from chest CT scans can predict the prognosis of NSCLC patients harboring *EGFR*-T790M mutation treated with second-line osimertinib. Our findings indicated that features extracted from the contrast-enhanced, rather than unenhanced, CT phase harbored satisfactory predicting power. Results from several other studies also supported that radiomic features from the contrast-enhanced CT scan showed superiority over the unenhanced CT phase in lung adenocarcinoma diagnosis, differentiating lung adenocarcinoma from squamous cell carcinoma and *EGFR*-related mutation prediction (31–33). However, there are also radiomic researches showing that features from the unenhanced phase had similar or even higher predictive ability over those from the contrast-enhanced chest CT (34, 35). Taking together, it can be implied that radiomic feature from distinct CT phases may have their own advantages when predicting different outcomes.

Several limitations exist in this study. Firstly, our study is a retrospective study using patients from a single center, and therefore, shortcomings associated with its study type cannot be ruled out. Besides, the sample size of the current study is small. Therefore, validations from investigations with larger samples in the future are needed. Furthermore, although 5-fold cross-validation was used in the present study, the predictive model developed in this study still needs further external validation. Finally, only a moderate performance was achieved in the calibration curves for model validation, suggesting that there is still room for improvement of the model.

## Conclusion

We firstly explored the prognostic value of radiomic features from chest CT scan in patients harboring *EGFR*-T790M mutation treated with second-line osimertinib therapy. Radiomic features extracted from contrast-enhanced chest CT scan exhibited great potential in prognostic evaluation. Prognostic models combing both radiomic features and clinical factors had a great performance in predicting patients’ outcomes. Our findings are helpful for predicting the prognosis of osimertinib treatment in NSCLC patients with EGFR-T790M and facilitating clinical decision-making.

## Data Availability Statement

The datasets presented in this article are not readily available because of the privacy of patient information. Requests to access the datasets should be directed to Z-gY, yangzg666@163.com.

## Ethics Statement

The studies involving human participants were reviewed and approved by the Ethics Committee of West China Hospital. Written informed consent for participation was not required for this study in accordance with the national legislation and the institutional requirements.

## Author Contributions

XT and YL: conception and design. XT, YL, W-fY, W-lQ, TP, Y-lG, and Z-gY: data collection and analysis. XT and YL: writing the manuscript. Y-lG and Z-gY: revising and editing the manuscript. All authors contributed to the article and approved the submitted version.

## Funding

This work was supported by the National Natural Science Foundation of China (No. NSFC30900360).

## Conflict of Interest

The authors declare that the research was conducted in the absence of any commercial or financial relationships that could be construed as a potential conflict of interest.

## Publisher’s Note

All claims expressed in this article are solely those of the authors and do not necessarily represent those of their affiliated organizations, or those of the publisher, the editors and the reviewers. Any product that may be evaluated in this article, or claim that may be made by its manufacturer, is not guaranteed or endorsed by the publisher.

## Supplementary Material

Supplementary Figure 1 The correlation of the 23 and 6 radiomic features extracted from the contrast enhanced and unenhanced CT scan after the feature selection by LASSO COX regression. Radiomic features extracted from the contrast enhanced CT were marked in red color. LASSO, Least absolute shrinkage and selection operator.
